# Low level off-road vehicle (ORV) traffic negatively impacts macroinvertebrate assemblages at sandy beaches in south-western Australia

**DOI:** 10.1038/srep24899

**Published:** 2016-04-28

**Authors:** Rebecca Davies, Peter C. Speldewinde, Barbara A. Stewart

**Affiliations:** 1 Centre of Excellence in Natural Resource Management, The University of Western Australia, PO Box 5771, Albany, WA 6331, Australia

## Abstract

Off-road vehicle use is arguably one of the most environmentally damaging human activities undertaken on sandy beaches worldwide. Existing studies focused on areas of high traffic volumes have demonstrated significantly lower abundance, diversity and species richness of fauna in zones where traffic is concentrated. The impact of lower traffic volumes is unknown. This study aimed to investigate the impacts of relatively low-level vehicle traffic on sandy beach fauna by sampling invertebrate communities at eight beaches located in south-western Australia. We found that even low-level vehicle traffic negatively impacts the physical beach environment, and consequently, the ability of many species to survive in this habitat in the face of this disturbance. Compaction, rutting and displacement of the sand matrix were observed over a large area, resulting in significant decreases in species diversity and density, and measurable shifts in community structure on beaches that experienced off-road vehicle traffic. Communities at impact sites did not display seasonal recovery as traffic was not significantly different between seasons. Given a choice between either reducing traffic volumes, or excluding ORV traffic from beaches, our results suggest that the latter would be more appropriate when the retention of ecological integrity is the objective.

Sandy beaches represent an important transition zone between marine and terrestrial environments^1^, where physical interactions between sediment and water movement determine beach morphology^2^. Beaches range from reflective (narrow, steep and high energy) to dissipative (wide, flat and low-energy), with most being an intermediate between these extremes^1^. The physical environment of sandy beaches is becoming intensely modified by anthropogenic activities, partly due to the advent of mass tourism and population growth in coastal areas^3^,^4^. Overall these factors are radically altering the ecology and morphology of coastal ecosystems, and increasingly, coasts are becoming sites of conflict over resource use between human demands and ecosystem preservation^5^. Threats to beach ecosystems arise from a range of stressors that span differing impacts, from global effects (e.g. sea level rise) to more localized ones (e.g. trampling of dune vegetation)^1^. Off-road vehicle (ORV) use is arguably one of the most environmentally damaging human activities undertaken on sandy beaches^6^,^7^ and can dramatically alter the physical properties of coastlines through the compaction, rutting and displacement of the sand matrix^8^. This vehicle traffic can substantially decrease organic matter in soils^9^ and can change the microclimate of the sand^5^.

This modification of sandy beach morphology is particularly problematic as biotic communities in these environments are primarily physically controlled, with ecosystem functioning, zonation and community structure primarily linked to beach morphological state^2^. In particular, community structure is correlated with sand particle size and beach face slope^10^,^11^, and species richness decreases from dissipative to reflective beaches^12^. Beach species are also generally not found in other environments as they often display unique adaptations to the dynamic system they inhabit^13^. Macroinvertebrate communities at sandy beaches are represented by most invertebrate phyla and are important components of both marine and terrestrial food webs. They predominately feed on algae and phytoplankton and can encompass scavenger, predator and filter feeder species^14^ as well as provide key food sources for higher order consumers such as fish and shorebirds^14^,^15^.

Investigations of the impacts of ORV use on sandy beach biota have focused either on indicator species^7^,^16^^–20^ or on macroinvertebrate assemblages^21–23^. Impacts on indicator species have been shown to be negative. For example, both ghost crabs (*Ocypode* species) and surf clams (*Donax deltoids*) had lower densities and decreased body sizes at beaches with ORV traffic^7^,^16^,^17^,^24^, with decreased home ranges^19^ and changed burrow architecture^18^ recorded for ghost crabs. Sheppard *et al.*^25^ found that the body mass index of the surf clam was 16% less at beaches open to vehicle traffic, and after 30 traffic passes, the burrowing ability of these clams was significantly impaired. Studies into the effects of ORV traffic on whole suites of species have been limited despite the fact that these taxa are vulnerable, as they generally inhabit zones where traffic is concentrated^21^,^22^,^23^. Existing Australian studies on the impacts of ORVs on beach morphology^22^ and macrobenthic assemblages^21^,^23^ in Queensland have been conducted in areas with high traffic volumes; these authors have reported 500–727 vehicles crossing their impact beaches per day, with as many as 5000 vehicles (South-East Queensland beaches) recorded in a single day during peak holiday periods and public holidays. As a result, ORV-impacted beaches had significantly lower abundance, diversity and species richness of macrobenthos species, particularly in the upper and middle zones where traffic was concentrated^23^. As well as the impacts on invertebrates, ORV use on beaches also has impacts on larger vertebrates, in particular nesting birds^26^,^27^ where ORV use can interfere with behaviour and/or disturb nests.

The impact of lower traffic volumes on macroinvertebrate communities inhabiting sandy beaches in Australia is unknown. One of the options available to managers aiming to decrease the deleterious environmental impacts of ORVs on sandy beaches would be to control access, and thus traffic volumes. For example, ORV access to Sodwana Bay in KwaZulu-Natal, South Africa is limited to 200 vehicles per day between 06:00 and 18:00 during weekends and holidays, with no access outside of these periods^20^. These restrictions have minimsed the impacts on fauna^20^. In order to determine threshold values of traffic volumes at which negative impacts occur for beach fauna in Australia, the impact of low-level traffic volumes should be further investigated. The beaches surrounding the south coast township of Albany, Western Australia provide a good opportunity for such a study, as this area has a mix of both ORV-impacted, and non-impacted beaches. Although formal traffic surveys have not been conducted for these ORV-impacted beaches, estimates of traffic volume based on tyre tracks, informal observations and consultation with beach users at four ORV-impacted beaches vary from 12–50 vehicles per day in summer to 7–40 vehicles per day in winter. In contrast studies in Queensland have quoted figures of approximately 300 vehicles a day^22^. This study aimed to investigate the impacts of this relatively low-level vehicle traffic on sandy beach fauna in south-western Western Australia. More specifically, we sampled both ORV-impacted and non-impacted beaches to (i) describe the habitat and sediment characteristics of these beaches, (ii) document ORV traffic distribution and impact on the physical environment of beach faces of ORV-impacted beaches, and (iii) investigate the invertebrate fauna response to this ORV traffic. This is the first study of the impacts of ORV traffic on macroinvertebrate fauna in south-western Australia, and is also the first to document impacts of relatively low-level traffic in Australia.

## Results

### Habitat and sediment characteristics

All the beaches studied had similar sediment properties and habitat characteristics for both sampling periods (ANOSIM, p = 0.1, summer: r = −0.177, winter: r = −0.208). Average sediment grain size varied from 149.9 to 295.8 microns, sediment moisture content ranged from 2.1–18.6% and sand fall velocity ranged from 1.74 to 4.21 cm/sec for all beaches (Table 1). Beach width ranged from 14.7 to 34.0 m, slope ranged from 2.8 to 10.3 degrees, average time between waves varied from 6.8–12.8 seconds and average wave breaker height was 0.70–1.25 m on the days sampled (Table 1). There were no significant differences in beach morphology between control and impact beaches as measured by the four beach indices during both summer and winter (ANOSIM, p = 0.1, summer: r = −0.115; winter: r = 0.073), and all beaches recorded fluctuations between low reflective and high intermediate morphological states. Beach characteristics remained similar throughout the study period, with only beach face slope showing significant differences between the seasons (ANOVA, p = 0.002, n = 16) as average slope in winter was considerably steeper than during summer (Table 1).

### Vehicle traffic

No tyre tracks were recorded during either season at control beaches. A total of 325 individual tyre tracks were mapped during the summer sampling period for ORV-impacted beaches, with Nanarup Beach having the highest number (110) and Gull Rock Beach, the least number of tyre tracks (47) (Table 2). Most of the impact beaches had an average number of tracks per linear metre of beach face above one, ranging from 0.53 tracks per meter (Gull Rock) to 1.58 tracks per meter (Cheynes Beach).

Off-road vehicle traffic was usually concentrated in the dry upper zone, ranging from 25% disturbance (Gull Rock Beach) to over 90% (Nanarup Beach) of this area (Table 2). Consequently this upper zone was considerably rutted, displaced and compacted. The deepest ruts occurred in the soft sand near the foredune and the widest ruts occurred lower down on the beach face towards the swash zone. Overall, ruts as deep as 26 cm (Gull Rock Beach) were recorded in the upper zone, although on the more naturally compacted sand of Cheynes Beach, deepest ruts were only 3 cm. Although there was an overall decrease in the number of tyre tracks observed in winter (total of 247) when compared to summer (total of 325), mean numbers of tyre tracks per beach for summer and winter did not differ significantly (ANOVA, p = 0.57, n = 8). Similarly, there were no significant differences between summer and winter in both average tracks per linear metre of beach face and the maximum number of tracks per metre recorded (ANOVA, p = 0.51 and 0.37 respectively, n = 8), indicating that ORV traffic in the Albany region was similar for both seasons at the time of sampling.

### Invertebrate fauna response

**Figure 1:**
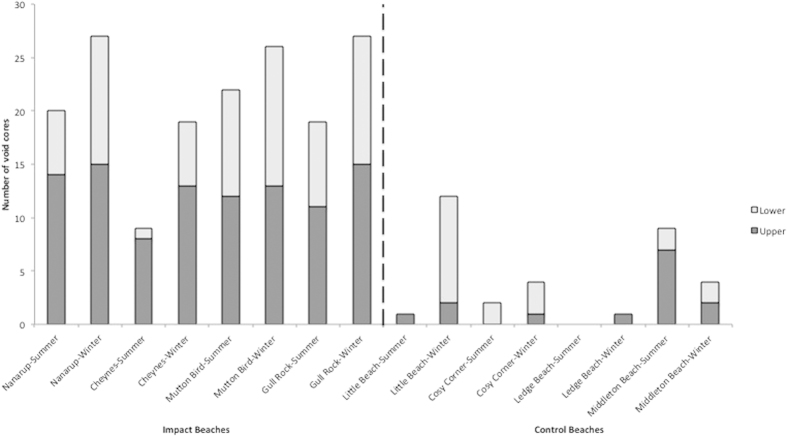
Number of levels sampled at the upper and lower zones of the eight sites which displayed no faunal occurrences(void samples). There were 30 levels excavated at each beach.

A large proportion of samples (42.1%) collected from both impact and control sites were devoid of fauna (Fig. 1). The control beaches had an average of 10% empty cores for both upper and lower zones. Ledge Beach had no empty cores, with fauna being collected from every level. Two control beaches had fauna present in every core taken in the upper levels in summer (Cosy Corner and Ledge Beaches). The ORV-impacted sites (mean of 10.56 void cores) had five times more void cores than the control beaches (mean of 2.06 void cores) for both seasons (ANOVA, p < 0:001, n = 16). For these sites, there were significantly more void cores in the upper (mean of 12.62 void cores) than in the lower (8.50 void cores) zone (ANOVA p = 0.026, n = 8). At least half of the cores taken from the upper zone at these four ORV-impact beaches were devoid of fauna, with Nanarup Beach (93.3% of cores from upper zone), Mutton Bird Beach (80%) and Gull Rock Beach (73.3%) having significantly high numbers of void cores in this zone. Although Cheynes Beach (53%) had a lower number of void cores collected from the upper zone, values were still comparatively higher than for the control beaches. Overall, there was a significant difference in void core numbers between the control and impact beaches for both the upper (impacted beaches: mean of 12.62 void cores; control beaches: 1.88 void cores; n = 8) and lower (impacted beaches: 8.50 void cores; control beaches: 2.25 void cores; n = 8) levels (ANOVA, p < 0:001 and p = 0.003 respectively, n = 120).

**Figure 2:**
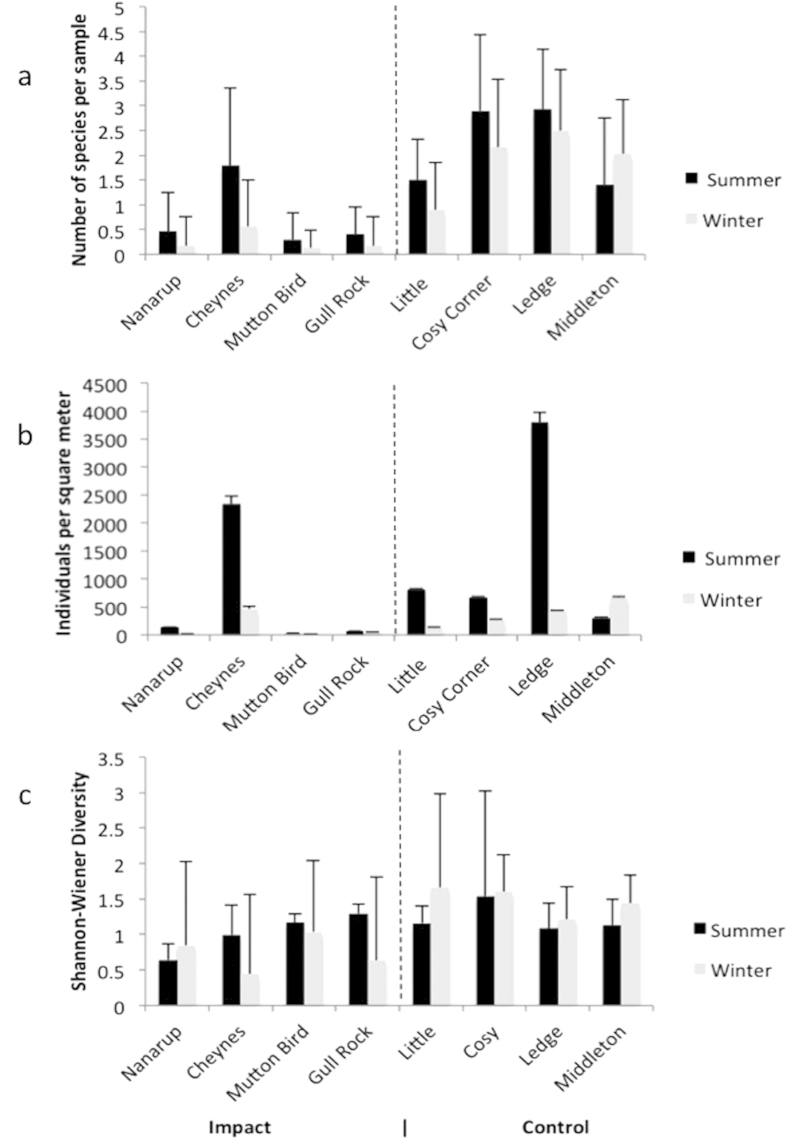
Differences in (a) Total abundances (individuals per square meter), (b) species density (number of species persample) and (**c**) species diversity (Shannon Wiener Diversity Index) between control and impact sites during both summer andwinter sampling.

For cores that contained fauna, 23 different species were recorded, with 4616 individuals sampled. Numerically, crustaceans dominated, representing 42% of individuals collected (12 species). Major crustacean groups were Isopoda (contributing 26.9% to the whole), Decapoda (8.5%) and Amphipoda (6.6%). Insects were the second most speciose group, with seven species recorded, however they contributed substantially less to the overall numbers, making up only 14.6% of the individual count. The single most abundant species was the mollusc Paphies elongate, with 1922 individuals collected over the entire sampling period. Overall, the ORV-impacted beaches demonstrated significantly lower species richness, species diversity (as measured by Shannon-Wiener Index) and abundance than control beaches (ANOVA, p < 0:001, n = 480) for both upper and lower zones (Fig. 2). Three-way ANOVA revealed significant differences for both category (impact vs. control) and beach (nested within category). There was also a significant two-way interaction between beach and season, and beach and zone (Table 3). In the upper zone, species richness was a mean of 1.83 (S.D. = 1.19) species per sample at the control beaches and 0.20 (S.D. = 0.53) species per sample at the impact sites. Similarly, species richness in the lower zone was 2.01 (S.D = 1.46) species per sample at the control beaches and 0.80 (S.D. = 1.17) species per sample at the impact beaches. The Shannon-Wiener diversity index for the upper zones of the control beaches was 0.39 (S.D. = 0.43) and 0.20 (S.D. = 0.53) for the upper zones of the impact beaches. Values for species diversity followed a similar trend for the lower zones (impact beaches: 0.80 (S.D. = 1.17); control beaches: 2.00 (S.D. = 1.46)). For the upper zones, macroinvertebrate abundance was considerably higher at control beaches (22.0 individuals per m^2^) than at impact beaches (1.7 individuals per m^2^). In contrast, abundance for the lower zones was similar (control beaches: mean of 36.8 individuals per m^2^; impact beaches: 24.2 individuals per m^2^).

Although NMDS analysis revealed considerable overlap among sites in terms of community structure, particularly in the lower zone, there were significant differences in community structure for the lower zone between control and impact beaches in summer (ANOSIM, p = 0.1, r = 0.222 and p = 0.1, r = 0.111 respectively, n = 240). In the upper zone, where ORV traffic was more frequent, community structure was significantly different between control and impact beaches for both summer and winter (ANOSIM, p = 0.1, r = 0.42 and p = 0.1, r = 0.434 respectively). These shifts in community structure were most strongly correlated to vehicle traffic indices and average time between waves, rather than a combination of environmental factors. For the upper zone, no single soil or beach habitat characteristic was more strongly correlated to changes in faunal composition than number of vehicle tracks, average % overall beach face disturbed or average tracks per metre. For the lower zone which is characterised by lower traffic intensity and swash-effects, shifts in community structure were more strongly correlated with average time between waves than any traffic index. This was closely followed by average percentage overall beach face disturbed, which indicates interplay between these characteristics in forming macrobenthic species composition.

## Discussion

This study clearly highlights that even low-level ORV traffic negatively impacts the physical beach environment, and con-sequently, the ability of many species to survive in this habitat in the face of this disturbance. Compaction, rutting and displacement of the sand matrix were observed over a large area, with shearing effects sometimes extending up to a depth of over 20 cm. As a result, communities on beaches subjected to traffic displayed significantly decreased species diversity and density, causing measurable shifts in community structure.

Our study also revealed three other key findings. Firstly, despite recommendations that ORV vehicles drive on the hard compacted sand of the lower zone^21^, ORV traffic was heavily concentrated in the upper zone, leading to a clear delineation of the beach face^23^,^28^. Secondly, impacts on faunal assemblages were greatest at the more intensely used upper zones with marked differences recorded in faunal abundances, species richness and density. Both zones displayed significant differences in species richness and species diversity among control and impact sites; however the differences were more noticeable for the upper than for the lower zone. This is in contrast to the study of Schlacher *et al.*^23^ who reported similar abundances, species richness and species diversity among impact and control beaches for lower zones in Queensland. It appears therefore that ORV impacts in the foreshore area (lower zone) are greater in south-western Australia than at other beaches studied. Schlacher *et al.*^23^ reported traffic numbers that were significantly higher than observed in our study, however, they found that 91% of this traffic traversed the upper and middle shore. In our study, occurrence of tyre tracks showed that ORV traffic was occurring at the lower zones at greater percentages than that reported by Schlacher *et al.*^23^ The most probable explanation of this differing traffic behaviour is that beaches in south-western Australia were much narrower (approximately 15–35 m) than those studied by Schlacher *et al.*^23^ (approximately 57–75 m), forcing many drivers to traverse a greater percentage of the beach face. Deep ruts were also found along the upper zone at narrow sections of beach and traffic may have needed to drive lower down towards the swash zone in order to negotiate around this rut. For the beaches studied in Queensland, the greater beach width meant it was probably unnecessary to drive along the swash zone, and thus the frequency of traffic and its impacts were not so severe in this region. Thirdly, our results suggest that ORV impacts are more ‘press’ than ‘pulse’ disturbances^29^, with the number of tyre tracks displaying no significant difference between the summer and winter months. Pulse disturbances are short and clearly delineated events that occur over a short period of time; in contrast press disturbances are ongoing and maintained at a constant level. Although south-western Australia has a tourist on-peak season in summer and less busy winter period, this was not reflected by vehicle usage along beaches near Albany. This ongoing pressure meant that seasonal recovery of communities was not apparent as the impact beaches consistently recorded significantly different abundances, species diversity and density, and void core counts across both seasons.

In conclusion, our results show that macroinvertebrate species at beaches with relatively low-levels of ORV traffic are as negatively affected as those at more intensively used beaches. This result has implications both globally and in south-western Australia, as sandy beaches continue to come under pressure from off road vehicle use. While completely excluding ORV traffic from beaches may not be feasible on all beaches some alternative management options are available such as restricting ORV use on stable beaches while allowing them on more dynamic beaches, having closed seasons on beaches to allow time for recovery and directing ORV use to low sections of the beach which are more dynamic rather than the higher sections which are more stable. Restricting access on narrower beaches may be more important than on wide beaches as the wider beaches allow more use of the lower parts of the beach. Given a choice between either reducing traffic volumes, or excluding ORV traffic from beaches, our results suggest that the latter would be more appropriate when the retention of ecological integrity is the objective.

## Methods

### Study area and sampling

**Figure 3:**
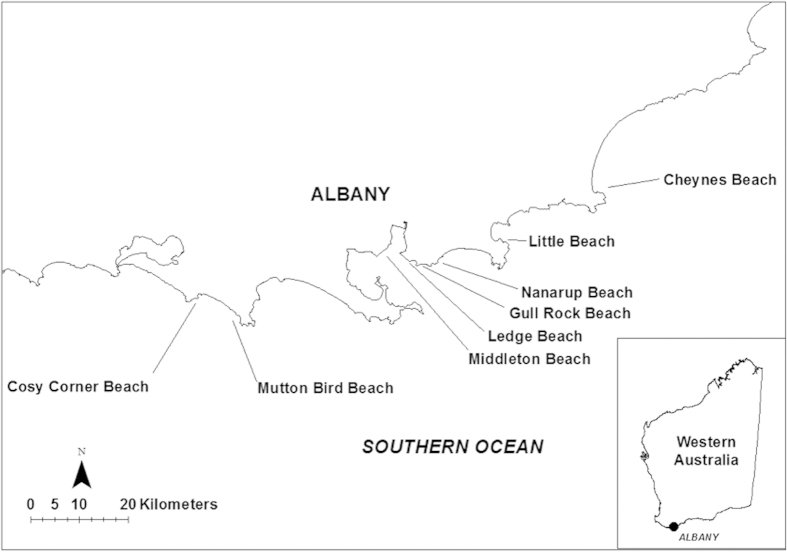
Location of study sites (Generated in ArcGIS 10.2).

Sampling was conducted at four beaches subjected to ORV traffic (impact beaches) and four without any vehicle use (control beaches). All eight beaches were located in south-western Australia near the city of Albany (Fig. 3). This region experiences a Mediterranean climate, with mild winters and hot, dry summers and the coast is open to the Southern Ocean. It has a mixed and mainly diurnal tidal regime, with the spring tidal range being less than 0.5 m^30^. The increasing use of the south-western Australian coast has led to significant pressures on the south coast marine environment. Off road vehicle use in the area has already been implicated in the decline of some shorebird species and during recent public consultation, ORV access to beaches was the issue of most concern^31^.

Each beach was sampled on two occasions, once during a period corresponding to high-intensity ORV use in the austral summer (February 2009 after peak tourist holidays), and once during winter when traffic volumes were low (August 2009). Benthic macroinvertebrates were sampled across three randomly placed transects perpendicular to the shoreline, with each transect positioned from the base of the foredune to the swash zone. Ten levels were sampled along each individual transect, with the upper levels (1–5) beginning at the foredune (‘upper zone’), and the lower levels (6–10) located closer to the water (‘lower zone’). At each level, a composite sediment sample was collected by combining three separate quadrats (25 cm deep, 30 × 50 cm) and then washed through a 1 mm mesh sieve. Fauna collected was preserved in 80% ethanol and later identified to the lowest possible taxon.

At the four impact beaches, the physical disturbances caused by ORV traffic was quantified by counting and measuring vehicle wheel ruts crossing each transect. As the ruts contained more than one tyre track, visual identification of different tyre patterns was used to estimate the number of tyre tracks in each rut. The percentage overall beach-face disturbed by vehicle traffic was also quantified along each transect. Vehicle ruts have been shown to be a good indicator of vehicle traffic with increase in the number of ruts associated with increased vehicle traffic^32^.

During both sampling periods, habitat characteristics were sampled at each beach. Sediment cores (25 cm diameter, 20 cm deep) were excavated at each level (1–10) along each transect. Sediment moisture content was determined in the laboratory by measuring the weight loss after drying to constant weight (105 degrees Celcius for 48 hours). Granulometry was ascertained by combining the sediment samples for each respective beach and dry sieving through a series of nested sieves (1000, 500, 250, 125 and 63 microns). Sediment statistics (average grain size, sorting, kurtosis and skewness) were calculated using the Folk and Ward method in the GRADISTAT software package^33^. Additional beach characteristics measured at each transect were width and slope (from foredune to swash zone), time between waves (in seconds) and wave breaker height. Beach morphological type (dissipative, intermediate or reflective) was determined using four different models: Dean’s parameter (D),Beach State Index (BSI), Beach State (BI) and the Beach Deposit Index (BDI).

### Statistical analysis

Significant differences in habitat characteristics, volumes of traffic (as measured by tyre tracks) and macroinvertebrate abundance, species density and species diversity (Shannon-Wiener Index) among sites and between seasons, were determined using one-way Analysis of Variance (ANOVA). Hierarchical three-way ANOVA utilized both raw and transformed (log(X + 1)) data. The variables included in this analysis were (i) category (impact vs control), (ii) beach (nested within category), (iii) season and (iv) zone. In a multivariate approach, significant differences among sites (based on habitat characteristics and beach indices) and community structure were determined using ANOSIM in the PRIMER (Plymouth Routines in Multivariate Ecological Research) statistical software package. Links between faunal composition and habitat characteristics (sediment and beach characteristics, as well as traffic variables) were analysed using BIO-ENV in PRIMER.

## Additional Information

**How to cite this article**: Davies, R. *et al.* Low level off-road vehicle (ORV) traffic negatively impacts macroinvertebrate assemblages at sandy beaches in south-western Australia. *Sci. Rep.* **6**, 24899; doi: 10.1038/srep24899 (2016).

## Figures and Tables

**Table 1:** Overview of ORV traffic distribution and impact along the four ORV affected beaches.

Season	Beach	No.tyretracks	No.tracks/metre	Overalldisturbed	Upper zonedisturbed	Deepestrut	Widestrut
Summer	Nanarup Beach	110	1.2–1.52	62.0%	92.5%	22 cm	45 cm
	Cheynes Beach	75	0.69–1.58	40.0%	75.0%	3 cm	27 cm
	Mutton Bird Beach	93	1.03–1.35	50.0%	80.0%	25 cm	29 cm
	Gull Rock Beach	47	0.46–0.60	42.5%	25.0%	26 cm	32 cm
Winter	Nanarup Beach	33	0.48–0.67	43.0%	60.0%	29 cm	22 cm
	Cheynes Beach	152	1.50–1.68	61.7%	26.6%	3 cm	28 cm
	Mutton Bird Beach	28	0.29–0.86	32.5%	60.0%	15 cm	32 cm
	Gull Rock Beach	34	0.46–0.52	36.7%	52.5%	23 cm	27 cm

**Table 2:** Hierarchical Analysis of Variance (ANOVA) summary comparing total abundance (individuals per meter squared),species density (number of species per sample) and species diversity (Shannon Wiener Diversity Index) across several temporaland spatial scales using transformed log(X + 1) data.

	DF	Total Abundance	Density	Species Diversity
Main effects				
Category (Impact vs Control)	1	<0.001	<0.001	<0.001
Beach (nested)	7	<0.001	<0.001	<0.001
Season	1	<0.001	<0.001	0.186
Zone	1	<0.001	<0.001	0.010
Two-way interactions				
Category × Season	1	0.133	0.320	0.015
Beach × Season	7	0.002	<0.001	0.002
Category × Zone	1	<0.001	<0.001	0.050
Beach × Zone	7	<0.001	<0.001	0.171
Season × Zone	1			
Three-way interactions				
Category × Season × Zone	1	0.966	0.146	0.308
Beach × Zone × Season	7	0.792	0.170	0.583
Residual	444			
Total	479			

**Table 3:** Summary of BIO-ENV analysis looking at matches in environmental variable with faunal composition.

	Upper ZoneMin-Max	Lower ZoneMin-Max
Sand Characteristics		
Sand Grain Size	0.103–0.106	0.056–0.058
Sorting	0.153–0.156	0.06–0.06
Kurtosis	0.133–0.134	0.052–0.053
Skewness	0.101–0.104	0.059–0.064
Sand Moisture Content	0.028	−0.033
Beach Characteristics		
Slope	0.065–0.066	0.066–0.069
Width	0.061–0.064	0.068–0.07
Average Wave Breaker Height	0.038–0.042	0.059–0.06
Average Time Between Waves	0.242–0.244	0.219–0.220
Vehicle Traffic		
Number of Tracks	0.503–0.504	0.176–0.178
Average Percentage Overall Disturbed	0.513–0.514	0.209–0.210
Tracks per meter	0.509–0.510	0.185–0.191

Values are correlation coefficient on characteristics that can best explain the differences between impact and control sites inmacroinvertebrate communities. Values are based on Spearman correlation coefficients and analyses was undertaken for bothraw data and log(X + 1) transformations.
